# Integration of Physical, Genetic, and Cytogenetic Mapping Data for Cellulose Synthase (*CesA*) Genes in Flax (*Linum usitatissimum* L.)

**DOI:** 10.3389/fpls.2017.01467

**Published:** 2017-08-23

**Authors:** Olga Y. Yurkevich, Ilya V. Kirov, Nadezhda L. Bolsheva, Olga A. Rachinskaya, Zoya E. Grushetskaya, Svyatoslav A. Zoschuk, Tatiana E. Samatadze, Marina V. Bogdanova, Valentina A. Lemesh, Alexandra V. Amosova, Olga V. Muravenko

**Affiliations:** ^1^Engelhardt Institute of Molecular Biology, Russian Academy of Sciences Moscow, Russia; ^2^Shemyakin-Ovchinnikov Institute of Bioorganic Chemistry, Russian Academy of Sciences Moscow, Russia; ^3^Institute of Genetics and Cytology, National Academy of Sciences of Belarus Minsk, Belarus

**Keywords:** gene mapping *Linum usitatissimum* L., genome, chromosome, *CesA* genes, BLAST analyses, FISH, rDNA

## Abstract

Flax, *Linum usitatissimum* L., is a valuable multi-purpose plant, and currently, its genome is being extensively investigated. Nevertheless, mapping of genes in flax genome is still remaining a challenging task. The cellulose synthase (*CesA*) multigene family involving in the process of cellulose synthesis is especially important for metabolism of this fiber crop. For the first time, fluorescent *in situ* hybridization (FISH)-based chromosomal localization of the *CesA* conserved fragment (KF011584.1), 5S, and 26S rRNA genes was performed in landrace, oilseed, and fiber varieties of *L. usitatissimum*. Intraspecific polymorphism in chromosomal distribution of KF011584.1 and 5S DNA loci was revealed, and the generalized chromosome ideogram was constructed. Using BLAST analysis, available data on physical/genetic mapping and also whole-genome sequencing of flax, localization of KF011584.1, 45S, and 5S rRNA sequences on genomic scaffolds, and their anchoring to the genetic map were conducted. The alignment of the results of FISH and BLAST analyses indicated that KF011584.1 fragment revealed on chromosome 3 could be anchored to linkage group (LG) 11. The common LG for 45S and 5S rDNA was not found probably due to the polymorphic localization of 5S rDNA on chromosome 1. Our findings indicate the complexity of integration of physical, genetic, and cytogenetic mapping data for multicopy gene families in plants. Nevertheless, the obtained results can be useful for future progress in constructing of integrated physical/genetic/cytological maps in *L. usitatissimum* which are essential for flax breeding.

## Introduction

Cultivated flax (*Linum usitatissimum* L., 2*n* = 2*x* = 30) is an annual self-pollinated crop widely grown for use in food production, industry, and medicine. Currently, the genome of this valuable multi-purpose plant is being extensively investigated. The genome size of *L. usitatissimum* cultivar (cv.) CDC Bethune was estimated at ∼373 Mb based on flow cytometry and draft sequencing of this genome was performed (Wang et al., 2012). A genetic map of flax based on SSR- and SNP-markers, five genes from the fatty acid biosynthesis pathway (*fad2A, fad2B, fad3A, fad3B*, and *dgat1*) and a phenotypic trait (seed coat color), was created (Cloutier et al., 2011). Also, a physical map of the genome of the flax cv. CDC Bethune, which consisted of 416 fingerprinted contigs spanning almost 100% of its genome, was developed (Ragupathy et al., 2011). Besides, genetic maps of three flax populations (CDC Bethune/Macbeth, E1747/Viking, and SP2047/UGG5-5) containing between 385 and 469 mapped markers were constructed (Cloutier et al., 2012). According to the linkage groups (LGs), consensus genetic and physical maps of flax were created (Cloutier et al., 2012; Kumar et al., 2015). However, this map has not been correlated to flax cytogenetic mapping developed earlier (Muravenko et al., 2003, 2009; Rachinskaya et al., 2011), and chromosomal localization of individual genes is still unexplored. Incorporation of physical, genetic, and cytological maps in one integrated map of the genome of *L. usitatissimum* is particularly important for investigation of genetic peculiarities of this valuable plant and further progress in flax breeding. Among all methods of physical mapping, only molecular cytogenetic techniques, such as fluorescent *in situ* hybridization (FISH), allow specific DNA sequences to be directly localized on mitotic and meiotic chromosomes. In this context, it can help their association with the LGs of genetic maps. For that purpose, highly conserved genes are usually mapped, and among these are cellulose synthase (*CesA*) genes of plants.

The complex multigene family of highly conserved *CesA* genes is believed to encode the glycosyltransferase enzymes which are involved in the process of cellulose synthesis in plants and play a significant role in gelatinous cell wall formation (Kawagoe and Delmer, 1997; Delmer, 1999; Robert et al., 2004; Taylor et al., 2004; Saxena and Brown, 2005). High expression levels of *CesA* genes observed in fast growing tissues of *L. usitatissimum* indicate that these genes are particularly important for this fiber crop (Gorshkova et al., 2005; Chantreau et al., 2015). Recently, 16 predicted *CesA* genes of flax were aligned with the well-defined *CesA* genes of *Arabidopsis* and *Populus* (Mokshina et al., 2014). All proteins encoded by *CesA*s genes include two plants conserved regions: P-CR1 and P-CR2 (Delmer, 1999; Richmond, 2000; Saxena et al., 2001; Doblin et al., 2002; Kumar and Turner, 2015; Kaur et al., 2016). The *CesA* genes can be identified by the P-CR sequence which is highly conserved in all plant *CesA*s (Doblin et al., 2002; Kumar and Turner, 2015; Kaur et al., 2016). Based on comparative studies of the nucleotide sequences of six flax *CesA* EST subunits (GenBank: EF409998–EF410000, EF214742–EF214744), the primers amplifying the 301 bp cDNA fragment of *CesA*-6 subunit (GenBank: KF011584.1) were designed (Grushetskaya et al., 2010). The alignment of the nucleotide sequence of the amplified *CesA*-6 fragment with the known sequences of the *CesA* genes of *Arabidopsis, Populus*, and *Eucalyptus* showed that the obtained KF011584.1 fragment comprised the region of the conserved P-CR2 domain of the *CesA* genes (Richmond and Somerville, 2000; Liang and Joshi, 2004; Ranik and Myburg, 2006).

In the present work, FISH-based chromosomal localization of the *CesA* conserved fragment (KF011584.1), 26S, and 5S rRNA genes was performed in karyotypes of one landrace, one oilseed, and two fiber varieties of *L. usitatissimum*. Using BLAST analysis, localization of KF011584.1, 45S, and 5S rRNA sequences on genomic scaffolds and their anchoring to the genetic map was conducted to align the obtained FISH results with the available data on physical/genetic mapping and whole-genome sequencing of flax.

## Materials and Methods

### Plant Material

Four *L. usitatissimum* varieties were studied: Braginskij kryazh (landrace), LM-98 (oilseed), Slavnyj-82 (fiber), and Belita (fiber). The seeds of Braginskij kryazh and LM-98 were obtained from the germplasm collection of All-Russian Flax Institute, Torzok, Russian Federation; the seeds of Slavnyj-82 and Belita were obtained from the collection of the Institute of Genetics and Cytology, National Academy of Sciences of Belarus, Minsk, Belarus.

### Chromosome Slide Preparation

For FISH, the modified technique of chromosome spread preparation from flax root tips was applied. The seeds were germinated in Petri dishes on moist filter paper at room temperature. Root tips (of 0.5 cm) were excised and treated overnight (16–20 h) in ice-cold water with 1 μg/ml 9-aminoacridine (Sigma, St. Louis, MO, United States) to harvest elongated chromosomes (Muravenko et al., 2003). After the pre-treatment, the root tips were fixed in ethanol:acetic acid (3:1) for 3–24 h at room temperature. Before squashing, the roots were transferred into 1% acetocarmine solution in 45% acetic acid for 15 min. The cover slips were removed after freezing in liquid nitrogen. The slides were dehydrated in 96% ethanol and then air dried.

### DNA Probe Preparation

26S and 5S rDNA probes were isolated from genomic DNA of *L. austriacum* as described previously (Yurkevich et al., 2013). The rDNA probes were labeled directly with SpectrumAqua (26S rDNA) and SpectrumRed (5S rDNA) fluorochromes (Abbott Molecular, Wiesbaden, Germany) according to the manufacturer’s protocol.

Fragments of the conserved domain B of the *CesA*-6 subunit (GenBank: KF011584.1) were obtained from cDNA of *L. usitatissimum* by PCR with primers *F_À6_302: 5*′*-TTATTGCTGTCCAGAGAGAG-3*′ and *R_A6_302: 5′-AGAACCATATACTGGCAAGA-3′* developed previously (Grushetskaya et al., 2010). These DNA fragments were cloned using pGEM-T Easy Vector System (Promega, Madison, WI, United States) in competent *Escherichia coli* cells of DHS*a* strain and isolated by the Plasmid DNA Isolation kit (Evrogen, Moscow, Russian Federation) according to the manufacturer’s protocols. Then, the cloned fragments were sequenced using Applied Biosystems 3730 DNA Analyzer and then labeled by Fluorescein Labeling Kit (MirusBio, Madison, WI, United States) according to the manufacturer’s protocols. Four microliters of 10 mg/ml sonicated salmon sperm DNA (Gibco BRL, New York, NY, United States) was added to each labeled probe mix which was precipitated with 100% ethanol and dissolved in 50 μl hybridization solution (50% deionised formamide, 10% dextran sulfate, 1% Tween-20, and 2× SSC). The concentration of the final DNA fragment was ≥20 (ng/μl). The probes were stored at –20°C before use.

### FISH Procedure

Before FISH procedure, chromosome slides were pre-treated with 1 mg/ml RNase A (Roche) in 2× SSC at 37°C for 1 h and then washed three times for 10 min in 2× SSC. The slides were dehydrated in a series of 70, 85, and 96% ethanol solutions and then air dried. The hybridization mixture (15 μl) containing 40 ng of each labeled probe was added to each slide. Coverslips were placed on the slides and sealed with rubber cement. Slides with DNA probes were co-denatured at 74°Ñ for 5 min, placed in a moisture chamber, and hybridized overnight at 37°C. After removing the coverslips, the slides were washed twice with 0.1× SSC at 44°C for 10 min, followed by two washes with 2× SSC at 44°C for 5 min and the final 5 min wash in 2× SSC at room temperature. Prior to detection, the slides were soaked in 4× SSCT (0.1% Tween-20 in 4× SSC) at room temperature for 3 min and then incubated in a detecting buffer (5% fat-free dry milk in 4× SSCT) at 37°C for 30 min. The slides were washed in 4× SSCT at room temperature for 3 min. In the case of the conserved domain B the *CesA*-6 subunit was labeled directly by Fluorescein Labeling Kit (Kreatech Biotechnology, Amsterdam, Netherlands), the fluorescent signal amplification using FITC-Alexa 488 antibodies (VectorLabs, Youngstown, OH, United States) was performed.

After incubation for 60 min at 37°C with the detection mixture, the slides were washed three times with 4× SSCT for 3 min each at room temperature, followed by a short rinse in PBS. The slides were dehydrated and air dried in the dark.

### DAPI-Banding

After FISH procedure, the slides were stained with 0.125 μg/ml 4′,6-diamidino-2-phenylindole (DAPI) (Serva, Heidelberg, Germany) dissolved in Citifluor anti-fade solution (UKC Chem. Lab., Canterbury, United Kingdom).

### Chromosome Analysis

Metaphase chromosome spreads were selected for analysis in accordance with the principles previously defined for small-sized chromosomes (Popov et al., 2001). In karyotypes, chromosomes were identified according to the cytological classification of *L. usitatissimum* developed previously (Muravenko et al., 2009). The slides were examined using an Olympus BX-61 epifluorescence microscope (Olympus, Tokyo, Japan). Images were captured with monochrome charge-coupled device camera (Cool Snap, Roper Scientific, Inc., Sarasota, FL, United States). Then they were processed with Adobe Photoshop 10.0 software (Adobe, Birmingham, AL, United States). At least 15 metaphase plates were investigated for each specimen.

### Localization of *CesA*, 26S, and 5S rRNA Genes on Genomic Scaffolds and Their Anchoring to the Genetic Map

To integrate obtained FISH results with the whole-genome sequencing data, BLAST analysis with 5S (X59854.1), 26S (EU307117.1) rDNA, and KF011584.1 sequences was performed against *L. usitatissimum* scaffolds^1^. All scaffolds with significant similarity (1e-5) to the query sequences were selected. To anchor these scaffolds with the LGs established by Cloutier et al. (2012), BLAST analysis with primer pairs used for the linkage map construction was performed against these flax scaffolds.

## Results

### DAPI-Banding and FISH with 26S and 5S rDNA for Chromosomal Identification of the Studied *L. usitatissimum* Varieties

**FIGURE 1:**
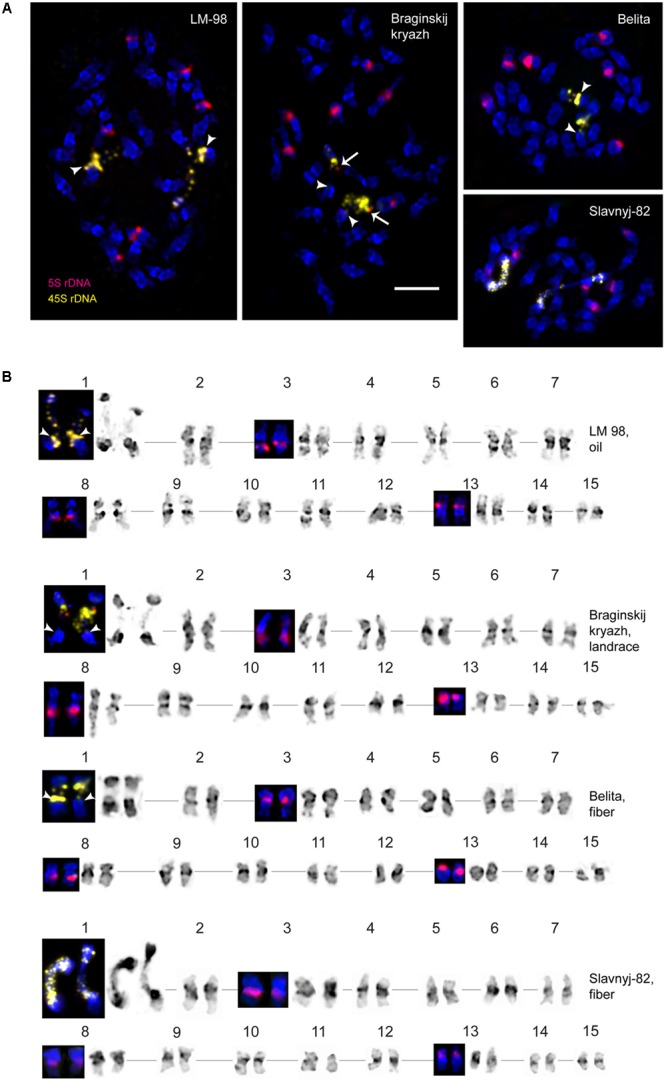
FISH-based localization of 26S (yellow) and 5S (red) rDNA on chromosomes of the studied *L. usitatissimum* varieties. **(A)** Metaphase spreads of LM-98 (oilseed), Braginskij kryazh (landrace), Belita (fiber), and Slavnyj-82 (fiber) after FISH; **(B)** karyograms [the same metaphase plates as in **(A)**] of these varieties after DAPI-banding (inverted image) and FISH (only chromosomes with the hybridizations sites are presented). The heads of arrows point to the polymorphic 26S rDNA sites (yellow) on satellite chromosome 1. The arrows point to the polymorphic 5S rDNA sites (red) on satellite chromosome 1. Bar – 5 μm.

**FIGURE 2:**
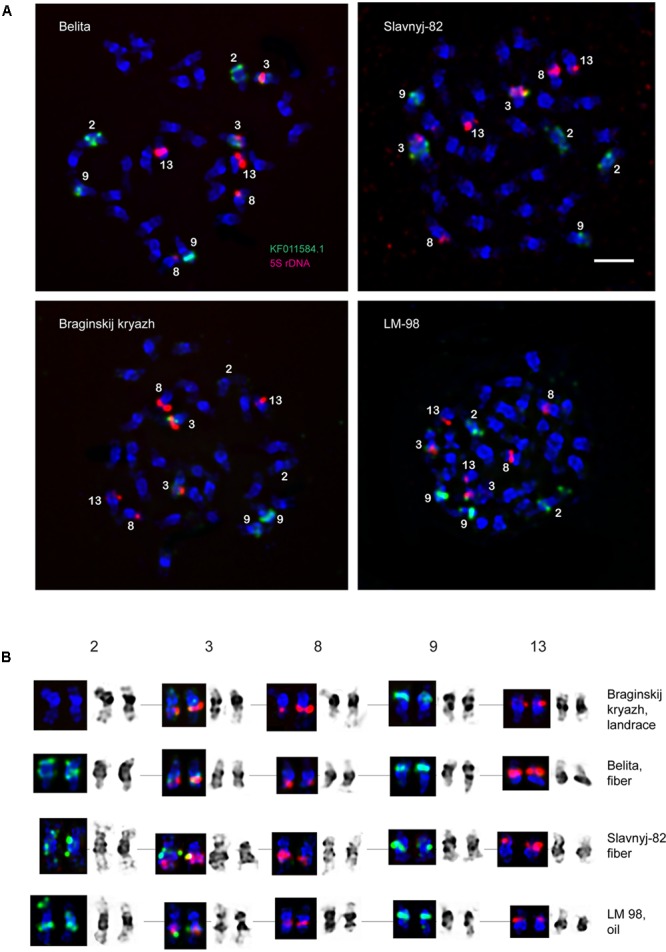
FISH-based localization of 5S (red) rDNA and the plant-conserved fragment (KF011584.1) of *CesA* genes (green) on chromosomes of *L. usitatissimum* varieties Belita, Slavnyj-82, Braginskij kryazh, and LM-98. **(A)** Metaphase spreads of Belita (fiber) Slavnyj-82 (fiber), Braginskij kryazh (landrace), and LM-98 (oilseed) after FISH; **(B)** the karyograms [the same metaphase plates as in **(A)**] after DAPI-banding (inverted image) and FISH (only chromosomes with the hybridizations sites are presented). Bar – 5 μm.

**FIGURE 3:**
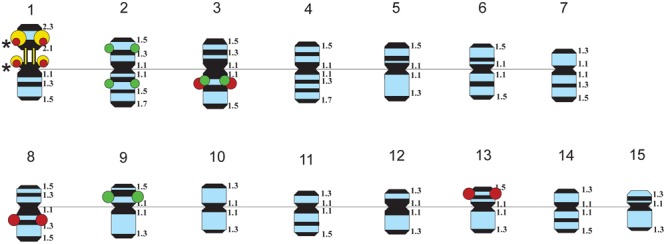
Idiograms of chromosomes in karyotypes of four studied *L. usitatissimum* varieties showing all possible positions of DAPI-bands (black segments), 26S rDNA (yellow), 5S rDNA (red), and the plant-conserved fragment (KF011584.1) of *CesA* genes (green). Asterisks indicate polymorphic 26S and 5S rDNA sites.

The karyotypes of all studied varieties consisted of 30 small-sized metacentric chromosomes (1–3 μm). DAPI-banding patterns of the karyotypes were chromosome-specific and mostly represented by large heterochromatic bands found in the pericentromeric regions and small bands detected in the telomeric and/or intercalary regions of the chromosomes (**Figures 1, 2**). DAPI-banding patterns allowed us to identify the homologous chromosome pairs in all studied karyotypes. In accordance with the cytological classification of chromosomes developed previously (Muravenko et al., 2009), the generalized idiogram of chromosomes of the studied *L. usitatissimum* varieties with account of all possibilities of DAPI-banding patterns, FISH-based localization of 26S, 5S rDNA, and *CesA* genes was constructed (**Figure 3**).

Fluorescent *in situ* hybridization analysis showed similar distribution of 26S rDNA sites in all studied varieties (**Figure 1**). One polymorphic (in size) 26S rDNA site was detected in the secondary constriction region of the satellite chromosome 1 (according to the cytological classification) involving the adjusting chromosomal areas (detailed in **Figure 1**). 5S rDNA loci were revealed in the proximal part of the long arm of chromosomes 3 (3L1.3) and 8 (8L1.3) as well as in the distal end of the short arm of chromosome 13 (13S1.3) in karyotypes of the studied varieties. In some plants of Braginskij kryazh variety, one small polymorphic 5S rDNA site (co-localized with 26S rDNA site) was observed on chromosome 1 (**Figure 1**).

### FISH Mapping of the *CesA* Gene Fragment

Localization of the conserved *CesA* gene sequence on metaphase chromosomes of the studied *L. usitatissimum* varieties was performed by FISH with the amplified *CesA*-6 fragment (KF011584.1) as a DNA probe (**Figures 2, 3**). In three varieties LM-98, Slavnyj-82, and Belita, sites of hybridization of the *CesA* genes were mapped on both arms of chromosomes 2 and also on chromosomes 3 and 9, but in Braginskij kryazh, sites of the *CesA* genes were revealed only on chromosomes 3 and 9 (**Figure 2**).

In chromosome 2, the polymorphic hybridization sites were localized in the distal end of the short arm (region 2S1.3) and in the proximal part of the long arm (region 2L1.3). In chromosome 3, the hybridization sites were mapped in region 3L1.2 of the long arm and also between the centromere and the region of localization of 5S rDNA site. In chromosome 9, bright hybridization signals were detected in the median part of the short arm (9S1.2) (**Figures 2, 3**).

### Anchoring of the *CesA* Genes to the Linkage Groups

Twenty-nine scaffolds and contigs with the length range from 109 to 2404031 bp showed similarity to the query sequence (*E*-value = 1e-5; coverage > 30%; identity > 50). Two scaffolds (157 and 1099) demonstrated the highest similarity (>98%) and query coverage (100%) while the other scaffolds showed lower similarity (<75%) and query coverage (<90%). To anchor these scaffolds to the LGs established by Cloutier et al. (2012), BLAST analysis with the primer pairs, used for the linkage map construction, was performed against the flax scaffolds. Nine of twenty-nine *CesA* possessing scaffolds could be anchored to the LGs by one to seven markers (**Table 1**). For some scaffolds (1186, 280, and 464), the markers belonged to different LGs indicating possible scaffold misassembles, errors in genetic mapping, or genome duplication regions. It was not possible to assign scaffolds 1186 and 464 to certain LG because markers were shared by different LGs. Nine anchored scaffolds were distributed along five LGs (1, 9, 11, 14, and 15). Based on the similarity, two top scaffolds (157 and 1099) were identical.

**Table 1:** Scaffolds with similarity to KF011584.1 anchored to the linkage groups (LGs) by markers.

n/n	Scaffold name	Contig length (bp)	Similarity to the FISH probe sequence	LG	Markers for anchoring of scaffolds to LGs	Position (cM)	Relative position on LG (%) Position ^∗^ 100/LG length
1	scaffold157	330988	99.3%; 299/301	1	Lu3231	130.76	76.9
2	scaffold1099	165476	98.9%; 299/301	1	Lu3231	130.76	76.9
3	scaffold103	720360	70.3%; 128/239	1	Lu2687	136.8	80.4
4	scaffold259	306757	73.6%; 198/269	9	Lu2168	64	67.7
5	scaffold57	1820724	72.4%; 176/243	11	lu512	55.1	64
				11	Lu785R224	55.8	65
				11	Lu3078	59	68.5
6	scaffold38	872253	74%; 199/269	14	Lu3043	17.6	23.14
				14	Lu3033	26.7	35.1
				14	Lu3046	27.85	36.6
7	scaffold360	1176022	74.8%; 190/254	15	Lu2695	52.46	87.6
				15	Lu2697	59.9	100
8	scaffold1376	440504	74.6%; 153/205	15	lu1007	46	76.8
9	scaffold280	2404031	70.6%; 173/245	15	lu271	6.3	42865
				15	Lu2931	7	42927
				15	lu510	8.7	42869
				15	Lu3186	11.9	20
				15	Lu3026	13.5	42877
				15	Lu3185	17.3	42975
				15	lu1163	22.2	37

**FIGURE 4:**
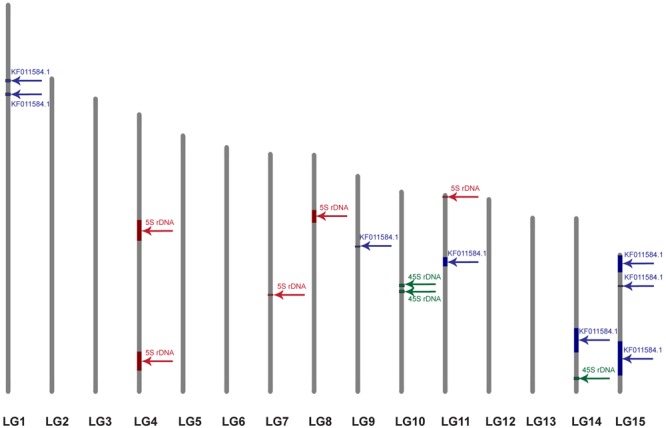
Localization of the scaffolds carrying 5S rDNA (red), 45S rDNA (green), and the plant-conserved fragment (KF011584.1) of *CesA* genes (blue) on the genetic map of *L. usitatissimum*. The framework of the genetic map was published earlier (Cloutier et al., 2012). The size of the colored box corresponds to the portion of the linkage group occupied by the scaffolds, which was calculated through the minimum and maximum positions of the markers belonged to the scaffolds.

For further integration of physical and genetic mapping, scaffolds carrying rRNA genes were identified by BLAST analysis with 5S (X59854.1) and 45S (EU307117.1) rDNA sequences of *L. usitatissimum*. We found 7 and 365 scaffolds with similarity to the 45S and 5S rRNA genes, respectively. *CesA* containing scaffolds possessed rRNA genes were not revealed. Five and three scaffolds with 5S and 45S rDNA, respectively, were anchored to the genetic map by at least one marker (**Figure 4**). Five 5S and three 45S rRNA gene contigs were located on four LGs (4, 7, 8, and 11) and two LGs (10 and 14), respectively.

## Discussion

The *CesA* multigene family encoding the glycosyltransferase enzymes plays a key role in the process of plant cellulose synthesis. These genes are especially important for bast fiber crops such as flax (*L. usitatissimum*) as the metabolism of fibers cells is oriented toward the extensive cellulose synthesis (Gorshkova et al., 2005; Chantreau et al., 2015). The problem of development of flax fibers is very important from a practical perspective because the elongation of the fiber cells as well as formation of the primary and secondary cell walls are directly associated with fiber quality, and also the yields of fiber flax varieties depend on the quantity and properties of fiber bundles (Gorshkova et al., 2005). The proteins encoded by plant *CesA*s genes are known to include two P-CRs (P-CR1 and P-CR2) *CesA*s which can be used for their identification (Richmond, 2000; Kumar and Turner, 2015; Kaur et al., 2016).

In the present study, using one of the *CesA* conserved fragment (P-CR2) as a FISH probe allowed us to localize the *ÑåsÀ* genes on *L. usitatissimum* chromosomes. Interestingly, FISH analysis did not reveal any peculiarities in *CesA* gene localization on chromosomes of fiber flax compared to the other studied *L. usitatissimum* varieties. The hybridization sites of the P-CR2 fragment of *CesA* genes were found on three pairs of chromosomes (2, 3, and 9). However, the hybridization signals observed on both arms of chromosome 2 were polymorphic. They were not found in the karyotype of the studied landrace (Braginskij kryazh) variety probably due to intraspecific variability of the copy number of the *CesA* genes. Some *CesA* genes could not be localized in case they were presented in few copies due to rather low resolution of FISH method (Jiang and Gill, 2006; Lamb et al., 2007; Karafiátová et al., 2013; Danilova et al., 2014). Therefore, the revealed sites of the *CesA* genes were apparently presented in flax chromosomes as multiple closely located isoforms or tandemly arranged copies.

BLAST analysis on localization of KF011584.1, 45S, and 5S rRNA sequences on genomic scaffolds and their anchoring to the genetic map of *L. usitatissimum* based on the data of Cloutier et al. (2011, 2012) showed that the studied fragment of *CesA* genes was located in eight loci within five LGs (1, 9, 11, 14, and 15). Genetic mapping studies of *Arabidopsis*, maize, and rice showed that the members of the *CesA* gene families were mostly spread across the genome although some genes were clustered together (Holland et al., 2000; Wang et al., 2010). Based on genetic mapping, *CesA* genes were detected in five barley chromosome pairs, in three *Arabidopsis* chromosome pairs, and in five maize chromosome pairs (Holland et al., 2000; Burton et al., 2004). *CesA* genes can be localized in one (shown for maize) or in both (in barley and wheat) chromosomal arms (Holland et al., 2000; Burton et al., 2004; Kaur et al., 2016). It has been shown that closely related *CesA* genes were often located in different chromosomes and the genes responsible for synthesis of the primary and secondary cell walls could be localized in one chromosomal region (Holland et al., 2000; Kaur et al., 2016). Unfortunately, the information on chromosome mapping of *CesA* genes is still rather limited, and we did not find any published studies on chromosome mapping of *CesA* genes in other plant species.

In the present work, 26S and 5S rRNA genes were used as chromosomal markers in FISH-based mapping of *CesA* genes. It is to be noted that polymorphism on distribution of 26S and 5S rRNA genes was previously described in karyotypes of different varieties of flax (Muravenko et al., 2003, 2009; Rachinskaya et al., 2011). We also detected polymorphism in distribution of 5S rDNA loci in karyotype of the landrace variety Braginskij kryazh. It was early shown that the number of 45S and 5S rRNA genes could vary considerably in *L. usitatissimum* (Goldsbrough and Cullis, 1981; Goldsbrough et al., 1982; Schneeberger et al., 1989). Based on restriction fragment length polymorphism (RFLP) and Random Amplification of Polymorphic DNA (RAPD) analyses, one site of rRNA genes and a specific subset of 5S rRNA genes were localized in one LG (13F) of flax hybrid lines (Oh et al., 2000). However, the association of rDNA genes with the LGs is still controversial probably due to the difficulties in genetic mapping of the multigene families (Ragupathy et al., 2011).

In this study, we identified scaffolds possessing 45S and 5S rRNA genes and anchored them to the six (4, 7, 8, 10, 11, and 14) LGs according to the linkage map of flax (Cloutier et al., 2011, 2012). According to our results, three loci of 45S rRNA genes were mapped within two LGs (10 and 14) though 26S rDNA sites were localized by FISH only in satellite chromosome 1. Also, five loci of 5S rDNA were mapped within four LGs (4, 7, 8, and 11). Considering the fact that 5S rRNA genes were localized by FISH on chromosomes 1 (polymorphic), 3, 8, and 13 (cytological classification), these chromosomes could probably be associated with the established LGs (4, 7, 8, and 11). Besides, the common LG for 26S and 5S rRNA genes was not found and this fact could be related to the intraspecific polymorphism in localization of 5S rDNA on chromosome 1. Our results may suggest that either the sensitivity of FISH method has a limited value for detection of the short rDNA sequences or there is significant DNA polymorphism between the cultivars used for the sequencing and the ones studied in the present work. Because of multiple rDNA localization on *L. usitatissimum* chromosomes, it is not currently possible to anchor FISH signals to the bioinformatically established loci. However, the obtained results can be used in the future as an additional source of information to produce an integrated genetic/physical map for this species.

Fluorescent *in situ* hybridization analysis showed that the studied fragment of *CesA* genes and 5S rDNA was localized very closely to each other in chromosome 3 (cytological classification). Besides, the *CesA* conserved fragment and 5S rRNA genes were co-localized only in one LG (11). This allowed us to assume that the *CesA* genes revealed on chromosome 3 could be anchored to the LG 11. Apart from chromosome 3, the *CesA* fragment was also localized in two chromosome pairs by FISH. However, the *CesA* sequence (seven loci) was anchored in four LGs (1, 9, 14, and 15). The observed discrepancies in the copy number and distribution of the *CesA* conserved fragment and 5S rRNA genes on the genetic and physical cytological maps can be explained by several reasons. First, the flax varieties studied in the present work and the cultivars used for genome sequencing might have copy number variation (CNV) of *CesA* genes. The information on the extent and distribution of CNVs of different genes in plant genomes is rather limited. Copy number variations were found for 30% of potato genes (Hardigan et al., 2016) and 10% of maize genes (Swanson-Wagner et al., 2010) as well as 2.2 Mb (2%) of *Arabidopsis thaliana* genome (Cao et al., 2011). Besides, CNVs of different genes were shown to play a part in regulation of the processes of plant adaptation to environmental stress (Iovene et al., 2013; Hardigan et al., 2016). It was early reported that the genome of *L. usitatissimum* possessed some labile DNA sequences (including rRNA genes) which can vary within a single generation when the plants are grown under specific environmental conditions (Oh and Cullis, 2003; Cullis, 2005). Second, due to rather low sensitivity of FISH (about 3000–10000 bp) (Jiang and Gill, 2006; Lamb et al., 2007; Karafiátová et al., 2013; Danilova et al., 2014), the studied P-CR2 fragment of *CesA* genes cannot be possible to visualize on mitotic flax chromosomes in case if it is presented in one or several copies.

Fluorescent *in situ* hybridization-based cytogenetic maps were integrated with genetic maps for a number of cultivated species: rice (Chen et al., 2002), melon (Gonzalez et al., 2010), grapes (Scalabrin et al., 2010), maize (Wei et al., 2009), cotton (Cui et al., 2015), and *Rosa wichurana* (Kirov et al., 2014, 2016). In the present study, for the first time, chromosomal localization of the highly conserved fragment belonging to the *CesA* multigene family, 5S, and 26S rRNA genes was aligned to the integrated genetic/physical map of *L. usitatissimum*. For construction of the integrated map of flax, the increase in the number of new sequence-based molecular and chromosome markers is needed. Our findings show the complexity of integration of physical, genetic, and cytogenetic mapping data for multicopy gene families in plants. Nevertheless, integration of physical, genetic, and cytological maps is essential for flax breeding progress, and the obtained results can be useful for future progress in constructing such a map for *L. usitatissimum*.

## Author Contributions

The present study was conceived and designed by OM, OY, IK, and VL. OY, OR, ZG, NB, AA, SZ, TS, and MB performed the experiments. OY, OM, NB, VL, IK, OR, SZ, TS, AA, MB, and ZG analyzed the data; bioinformatics analysis was provided by IK. OY, OM, IK, VL, OR, ZG, NB, SZ, TS, MB, and AA participated in preparing and writing the manuscript. OM, OY, VL, AA, IK, ZG, OR, SZ, TS, MB, and NB performed the analysis with constructive discussions. All authors contributed to revising the manuscript. All authors have read and approved the final manuscript.

## Conflict of Interest Statement

The authors declare that the research was conducted in the absence of any commercial or financial relationships that could be construed as a potential conflict of interest.

## Footnotes

1 http://www.phytozome.net
